# Cross-Reactive Results in Serological Tests for Borreliosis in Patients with Active Viral Infections

**DOI:** 10.3390/pathogens11020203

**Published:** 2022-02-03

**Authors:** Iwona Wojciechowska-Koszko, Paweł Kwiatkowski, Monika Sienkiewicz, Mateusz Kowalczyk, Edward Kowalczyk, Barbara Dołęgowska

**Affiliations:** 1 Department of Diagnostic Immunology, Pomeranian Medical University in Szczecin, Powstancow Wielkopolskich Av. 72, 70-111 Szczecin, Poland; pawel.kwiatkowski@pum.edu.pl; 2 Department of Pharmaceutical Microbiology and Microbiological Diagnostic, Medical University of Lodz, Muszynskiego St. 1, 90-151 Lodz, Poland; monika.sienkiewicz@umed.lodz.pl; 3 Babinski Memorial Hospital, Aleksandrowska St. 159, 91-229 Lodz, Poland; mateuszjerzykowalczyk@gmail.com; 4 Department of Pharmacology and Toxicology, Medical University of Lodz, Zeligowskiego St. 7/9, 90-752 Lodz, Poland; edward.kowalczyk@umed.lodz.pl; 5 Department of Laboratory Medicine, Pomeranian Medical University in Szczecin, Powstancow Wielkopolskich Av. 72, 70-111 Szczecin, Poland; barbara.dolegowska@pum.edu.pl

**Keywords:** Lyme disease, diagnostics, serological tests, antibodies, cross-reactivity, active viral infections

## Abstract

Currently, serological tests for Lyme disease (LD), routinely performed in laboratories following the European Concerted Action on Lyme Borreliosis recommendations as part of two-stage diagnostics, are often difficult to interpret. This concerns both the generation of false positive and negative results, which frequently delay the correct diagnosis and implementation of appropriate treatment. The above problems result from both morphological and antigenic variability characteristics for the life strategy of the spirochete *Borrelia burgdorferi* sensu lato, a complicated immune response, and imperfections in diagnostic methods. The study aimed to check the reactivity of sera from 69 patients with confirmed infection with Epstein–Barr virus (EBV), cytomegalovirus (CMV) and BK virus (BKV) with *Borrelia* antigens used in serological tests: indirect immunofluorescence (IIFT), enzyme-linked immunosorbent (ELISA) and immunoblot (IB). In the group of patients infected with EBV, the highest percentage of positive/borderline anti-*Borrelia* IgM and IgG results was obtained in the following tests: IIFT (51.9% for IgM, 63.0% for IgG), ELISA (22.2% for IgM, 29.6% for IgG) and IB (11.1% for IgM, 7.4% for IgG). In the group of CMV-infected patients, the highest percentage of positive/borderline anti-*Borrelia* IgM results were obtained in the following tests: IB (23.1%), IIFT (15.4%) and ELISA (7.7%), while in the IgG class in the IIFT (15.4%), IB (11.5%) and ELISA (3.9%) tests. In the group of patients infected with BKV, the highest percentage of positive/borderline anti-*Borrelia* IgM results was obtained in the following tests: IIFT (25.0%), IB (25.0%) and ELISA (3.9%), and in the IgG class in the tests: IB (50.0%), IIFT (6.2%) and ELISA (6.2%). The native flagellin (p41) and OspC proteins were the most frequently detected *Borrelia* antigens in all studied groups of patients in both classes of antibodies. Similar to other authors, the study confirmed the fact that serological tests used in the diagnosis of LD have a high potential to generate false positive results in patients with active viral infections, which may be related to cross-reacting antibodies appearing during the most common polyclonal activation of T/B lymphocytes, activated by viral superantigens.

## 1. Introduction

Lyme disease (LD) is a multi-organ anthropozoonosis caused by *Borrelia burgdorferi* sensu lato. Due to a varied clinical picture and non-specific symptoms, serodiagnosis of this disease is subject to the unified guidelines imposed by the European Concerted Action on Lyme Borreliosis (EUCALB) [1,2,3,4,5,6,7,8,9,10]. They relate to the two-stage method—the first stage includes the enzyme-linked immunosorbent (ELISA) or indirect immunofluorescence (IIFT) screening assays, and the second stage is a verification of positive/borderline results using the immunoblot (IB) method.

Unfortunately, at present, the interpretation of the obtained results using serological tests is problematic due to the generation of a large percentage of false positives or false negatives [6,9,11,12,13,14,15]. For this reason, the diagnosis should be based mainly on the clinical picture and history of tick exposure of the patient. Due to this fact, it should be remembered that results of laboratory tests only help to target and confirm it. In accordance with the EUCALB recommendations, laboratory tests are required to make a diagnosis and implement treatment in patients without erythema migrans [6,14,16]. The appearance of false positive results may be related to many factors: low specificity of serological tests, cross-reactions occurring during other bacterial and viral infections when polyclonal activation of T/B lymphocytes is observed and the occurrence of hypergammaglobulinemia [17]. It has been observed that the so-called superantigens, which may include, among others, Epstein–Barr virus (EBV), cytomegalovirus (CMV) and BK virus (BKV), are capable of polyclonal activation of T/B lymphocytes [17]. On the other hand, false negative results may occur if the test was performed too early or too late. Moreover, immune complexes (ICs), which may appear in massive and acute infections, may also contribute to false negative results [4,18,19]. These infections make free antibodies bind to bacterial antigens, making screening tests unable to detect specific immunoglobulins. Currently, research into methods for detecting immunoglobulins in complexes is underway, but they are not yet commercialised, e.g., enzyme-linked M capture IC biotinylated antigen (EMIBA) [18] or the EliSpot C6 Lyme assays [19].

The study aimed to check the reactivity of sera from 69 patients with confirmed infection with EBV, CMV and BKV with *Borrelia* antigens used in serological tests: IIFT, ELISA and IB.

## 2. Results

### 2.1. Result Analysis of EBV, CMV and BKV Presence with qPCR and Serological Methods

In all patients infected with EBV, CMV and BKV, the number of viral copies per mL of serum ranged between 560 and 17,800,568, 1078 and 85,093,861 and 920 and 39,976,524, respectively (Table 1). No copies of the analysed viruses were found in the serum of healthy individuals (control group).

Regarding the xMap Luminex test used to assess the specificity of anti-EBV antibodies, their all four types were identified: VCA IgM (12/27; 44.4%), EAD IgG (17/27; 63.0%), VCA IgG (27/27; 100.0%) and NA-1 IgG (16/27; 59.3%). On the other hand, the anti-CMV ELISA test detected IgM and IgG antibodies in 22/26 (84.6%) and 26/26 (100.0%) of patients, respectively.

Concerning the control group, the following antibodies were detected: anti-VCA IgG (1/20; 5.0%), anti-NA1 IgG (5/20; 25.0%) and anti-CMV IgG (7/20; 35.0%).

Detailed results of the determination of EBV, CMV and BKV copy numbers, as well as anti-EBV and anti-CMV serological analysis in the experimental and control groups, are summarised in the Supplementary Materials (Tables S1–S4).

### 2.2. Analysis of Anti-Borrelia IgM and IgG Antibodies Presence with Serological Methods

Table 2 presents results concerning the percentage and the number of positive/borderline and negative results of anti-*Borrelia* obtained in IgM and IgG class using the ELISA, IIFT and IB methods in the three analysed groups of patients with an active viral infection. Detailed data are summarised in the Supplementary Materials (Tables S1–S3).

In the group of patients infected with EBV, the highest percentage of positive/borderline anti-*Borrelia* IgM and IgG results was obtained in the following tests: IIFT (51.9% for IgM, 63.0% for IgG), ELISA (22.2% for IgM, 29.6% for IgG) and IB (11.1% for IgM, 7.4% for IgG). A statistically significant difference was only found between results of the following tests: IIFT/ELISA (*p* = 0.0473) and IIFT/IB (*p* = 0.00276) for IgM, as well as IIFT/ELISA (*p* = 0.0281) and IIFT/IB (*p* = 0.00003365) for IgG.

In the group of CMV-infected patients, the highest percentage of positive/borderline anti-*Borrelia* IgM results were obtained in the following tests: IB (23.1%), IIFT (15.4%) and ELISA (7.7%), while in the IgG class in the tests: IIFT (15.4%), IB (11.5%) and ELISA (3.9%). No statistically significant differences were found in CMV-infected patients.

In the group of patients infected with BKV, the highest percentage of positive/borderline anti-*Borrelia* IgM results was obtained in the following tests: IIFT (25.0%), IB (25.0%) and ELISA (6.3%). These results were not statistically significant. However, the highest percentage of positive/borderline results in the IgG class was obtained in the tests: IB (50.0%), IIFT (6.3%) and ELISA (6.3%). A statistically significant difference was found only between the results of the IB/ELISA and IB/IIFT tests (*p* = 0.0155).

There were two positive IgM anti-*Borrelia* results in the control group, two IgG results using the IIFT method and one borderline IgG result using the ELISA method. Detailed results of determination of anti-*Borrelia* antibodies in the IgM and IgG class, with the application of serological tests used in control group patients, are summarised in the Supplementary Materials (Table S4).

### 2.3. Verification of the Presence of Anti-Borrelia IgM and IgG Antibodies with IB

The most frequently detected anti-*Borrelia* IgM and IgG antibodies were the native flagellin (p41) and OspC proteins.

In the group of patients infected with EBV and CMV, IgM directed against *B. afzelii* flagellin (8/27, 29.6% for EBV-positive patients; 3/26, 11.5% for CMV-positive patients) and *B. garinii* OspC (3/27, 11.1% for EBV-positive patients; 6/26, 23.1% for CMV-positive patients) was most often detected. On the other hand, in patients infected with BKV, the most frequently detected IgM antibodies were anti-OspC *B. afzelli* and anti-OspC *B. garinii*, which remained at a similar level (3/16, 18.8%). In all EBV-, CMV- and BKV-infected patients, IgG directed against *B. garinii* flagellin was most frequently detected (10/27, 37.0% for EBV-positive patients; 11/26, 42.3% for CMV-positive patients; 15/16, 93.8% for BKV-positive patients).

**Figure 1:**
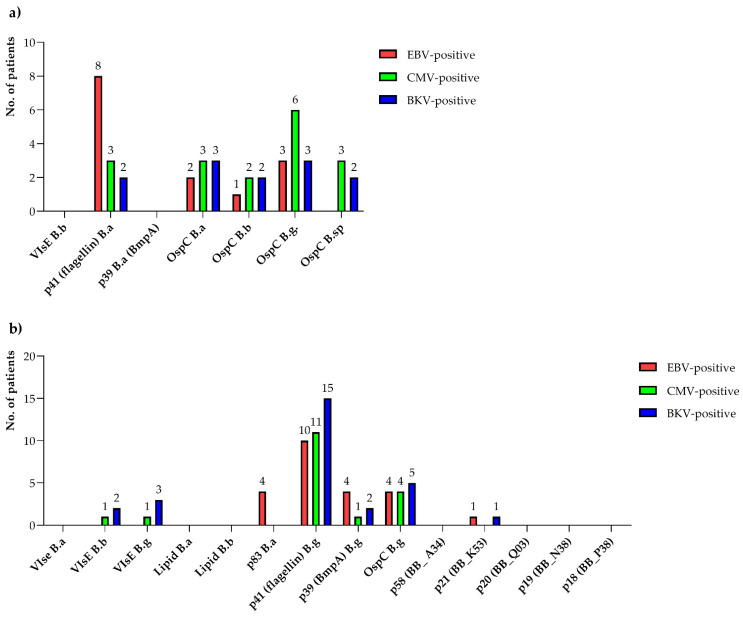
Number of patients with positive/borderline results of anti-*Borrelia* IgM (**a**) and IgG (**b**) antibodies obtained by immunoblotting for patients infected with the Epstein–Barr virus (EBV), cytomegalovirus (CMV) and BK virus (BKV): B.a, *Borrelia afzelii*; B.b, *Borrelia burgdorferi*; B.g, *Borrelia garinii*; B.sp, *Borrelia spielmanii*.

The specificity of detected anti-*Borrelia* IgM and IgG antibodies with the IB method in patients of the experimental group is presented in Figure 1, while detailed data are summarised in the Supplementary Materials (Tables S1–S3).

Concerning the specificity of anti-*Borrelia* antibodies with the IB test, no positive results were obtained in the control group in the IgM and IgG class. Detailed results of the determination of anti-*Borrelia* antibodies in the IgM and IgG class with the application of serological tests used in patients of the control group are summarised in the Supplementary Materials (Table S4).

## 3. Discussion

Currently, in Europe, it has become a common standard to use two-stage LD serodiagnosis following the EUCALB guidelines. In the first step, highly sensitive indirect screening methods such as IIFT or ELISA are used. Subsequently, borderline or positive results or both are verified with confirmatory tests characterised by high specificities, such as Western blot (WB) or IB. The latter method utilises recombinant antigens, which reduces the likelihood of, for example, cross-reactions and is characterised with slightly higher specificity and sensitivity. The IB method, being more advantageous than the WB method, is more preferable. Hence, there is a growing tendency to use it rather than the WB method. Studies conducted by Fawcett et al. [20] confirm that a 4% increase in specificity contributes to 95% sensitivity of the IB method and 76% sensitivity of the WB method. Other scientists reported similar observations [21,22,23]. In our research, the sensitivity of the IIFT test for the IgM class, as recommended by the manufacturer, was 80% and 95% for the IgG class. On the other hand, the sensitivity of the ELISA test ranged from 89 to 96% and was correlated with the disease stage—lower values are found in stage I and higher values in stage II. However, the EUROLINE RN-AT IB test, testing IgM antibodies, demonstrated 94.9% specificity and 93% sensitivity. However, for the IgG class, the test showed 90.3% specificity and 95.6% sensitivity.

Our research confirmed the fact that the serological screening tests used in the diagnosis of LD have a significant potential to generate false positive results in patients with active viral infections, which may be related to the cross-reactions occurring during the most common polyclonal activation of B cells by viral superantigens [17,23]. The false positive results obtained in the current study indicate the presence of LD in patients with a confirmed viral infection, while, in fact, these patients do not demonstrate clinical symptoms. It is important to bear in mind that individuals from LD endemic regions may have laboratory positive results but without clinical signs of LD. In such cases, patients with an active viral infection may appear to be “true false positives”, demonstrating non-specific cross-reactions in serological tests. It should also be pointed out that these tests may generate false negative results, as indicated in previous studies [5,10,13,24]. In this case, patients with LD symptoms are negative in diagnostic tests. Dattwyler et al. [24] concluded that the occurrence of chronic LD in patients cannot be ruled out due to negative results obtained in serological diagnostic tests. This confirms a specific blastogenic T cell response to *B. burgdorferi* in seronegative patients with clinical symptoms of chronic LD. Currently, in the case of ambiguous patients, high hopes are associated with research based on the detection and quantification of surface membrane proteins released by *Borrelia* spirochetes as a result of damage to their cell membrane caused by the innate immune system in serum or damage to other biological material by mass spectrometry or immunological electron microscopy [25,26,27].

In the current study, in the experimental group infected with EBV, the highest percentage of positive results for the detection of anti-*Borrelia* antibodies was recorded in the following tests: IIFT, ELISA and IB. On the other hand, in the group of patients infected with CMV and BKV, the highest number of positive results was obtained in the IB test, with a tendency to generate positive results in the IgM class. The higher percentage of positive results obtained in the IgM class rather than in the IgG class may result from lower avidity of this class of antibodies, which increases the probability of generating cross-reactions. Similar results concerning the level of anti-*Borrelia* IgM antibodies in patients infected with EBV and CMV were obtained by Goossens et al. [11] and Plewik et al. [28]. Other researchers also observed false positive results in anti-*Borrelia* serological tests in patients infected with other pathogens. Strasfeld et al. [29] reported cases of false positive results obtained in ELISA tests for IgM antibodies in patients with herpes simplex virus type 2 infections. Garment et al. [30] obtained a false positive result in the IgM class in anti-*Borrelia* ELISA and the WB test in a patient with thyroiditis, with positive results of IgG anti-CMV and anti-EBV antibodies.

Cross-reactions in serological tests are related to the type of antigens used in the test. Various literature sources indicate that during *Borrelia* infection in patients with suspected infection, an early immune response against the flagellin protein (p41) and the OspC protein (p23–25) is accompanied by an increase in the IgM level [31]. Additionally, antibodies to antigens such as p37, p35 and, very rarely, p39 (BmpA) can be detected. Then, an increase in the IgG antibodies level heralds a late immune response. At first, antibodies against VIsE, p39 (BmpA) and p58 proteins, and in the course of the disease, antibodies against many other antigens such as p83/p100, p93, p53, p43, BmpA, p30, p21 (BB_K53), Osp17 and p14 appear [4,32]. These IgG antibodies often cross-react with other pathogens such as *Treponema pallidum*, *Helicobacter pylori*, *Rickettsia* spp., *Ehrlichia* spp., human immunodeficiency virus 1 and herpes viruses [6]. On the other hand, antigens specific for *Borrelia* spirochetes include proteins such as OspA, OspC, BmpA, p93 and p83/p100 [4].

In the current study, in patients belonging to all three groups, IgM antibodies against the following antigens were found in the EUROLINE RN-AT IB test: the *B. afzelii* (p41) flagellin antigen and the OspC antigen belonging to three or four genotypes (in the case of EBV-positive patients, we detected anti-OspC *B. garinii*, *B. afzelii* and *B. burgdorferi* sensu stricto; in the case of CMV and BKV, anti-OspC *B. garinii*, *B. afzelii*, *B. burgdorferi* sensu stricto and *B. spielmanii*). In addition, Ma et al. [33] described that in patients with syphilis, a high percentage of positive results for the p41 antigen (75%) in the WB IgM test was noted.

In turn, in the IgG antibody class, the following positive reactions were observed in all three groups: OspC *B. garinii*, p39 (BmpA) *B. garinii* and p41 (flagellin) *B. garinii*. In addition, antibodies against VlsE *B. garinii* and *B. burgdorferi* sensu stricto were detected in the BKV and CMV group of patients, while antibodies against *B. garinii* p21 (BB_K 53) antigen were detected in the EBV and BKV groups. In EBV-positive patients, the *B. afzelii* anti-p83 antibodies were only detected. Similar results were obtained by Ma et al. [33], who reported that 10% of sera of patients suffering from syphilis positively reacted with the p39 (BmpA) antigen. In contrast, Simpson et al. [34] found a lack of cross-reactions between the p39 (BmpA) antigen and the sera of patients with syphilis.

In our research, in the case of the IB test, the most frequently detected antigens in all three groups of tested patients in both analysed classes of antibodies were native proteins: p41 (flagellin) and the OspC antigen. As for anti-p41 antibodies within all the studied groups of patients, a higher percentage of positive results in the IgG class rather than in the IgM class was observed. Fawcett et al. [20] obtained similar results, which may be contributed by the fact that they used the native form of the p41 (flagellin) protein in their own tests, which, according to literature, has a more significant potential to cross-react with antibodies than the recombinant form. Studies by Rasiah et al. [35] indicate that the inner fragment of flagellin (p14) shows higher specificity in ELISA and IB tests compared with the p41 protein.

Application of both the native p41 (flagellin) protein and the native OspC protein in the IB test increased the number of positive results within the studied groups of patients. According to the literature, this protein in its native form is more sensitive but less specific than its recombinant form [36]. Similar results were obtained in the study by Goossens et al. [11].

Summing up, it should be remembered that an important aspect in the diagnosis of LD is the use of specific borrelial antigens. It is important for the determination of LD aetiology, and it can minimise cross-reactivity, especially in patients with an active viral infection. This is indicated by the significant statistical difference in positive results obtained in our research in the group of BKV-infected patients between screening and confirmations tests.

## 4. Materials and Methods

### 4.1. Patients

The study was conducted in 2012–2017. It included 69 patients (experimental group) hospitalised in the Department of Nephrology, Transplantology and Internal Diseases of the Independent Public Clinical Hospital No. 2 of the Pomeranian Medical University in Szczecin (Poland) with confirmed viral infections with EBV (*n* = 27), CMV (*n* = 26) and BKV (*n* = 16).

The patients were initially qualified for the study based on clinical symptoms and positive results of genetic tests ordered by the referring physician. Depending on the symptoms, a molecular test was performed to detect EBV, CMV or BKV DNA using the real-time PCR (quantitative PCR, qPCR) method. After this stage, the patients were interviewed for possible contact with the tick, autoimmune diseases and syphilis. Then, sera seroreactivity was assessed to detect specific IgM and IgG anti-EBV antibodies using the xMap Luminex method and the anti-CMV method, utilising the ELISA method. With regards to patients infected with the BKV virus, only the molecular test was performed due to a lack of commercial tests for detecting specific antibodies. After this stage, all sera were tested for LD by IIFT, ELISA and IB tests in two classes of antibodies—IgM and IgG.

The control group consisted of 20 healthy volunteers. Genetic tests were performed in all healthy individuals to exclude infection with EBV, CMV and BKV. The seroreactivity of the sera was also assessed for specific IgM and IgG anti-EBV antibodies using the xMap Luminex method and the anti-CMV method, utilising the ELISA method. Then, the specificity of antibodies towards *Borrelia* was assessed in the study group with the use of three serological techniques. An interview was conducted to collect data from all participants qualified for the study. Both the experimental and control groups had no history of contact with the tick and no symptoms indicative of LD. An autoimmune background and infection with syphilis were also excluded.

### 4.2. Detection of EBV, CMV and BKV with the qPCR Method

Sera from 27, 26 and 16 experimental patient groups were tested with Real-Time PCR GeneProof Epstein–Barr Virus PCR Kit, Real-Time PCR GeneProof Cytomegalovirus PCR Kit and GeneProof BK/JC Virus PCR Kit, respectively, according to the manufacturer’s protocols. All tests were purchased from GeneProof (Brno, Czech Republic).

Results are reported as virus copy number/mL. The quantitative results of CMV, BKV and EBV were analysed with the croBEE Real-Time PCR System computer programme from GeneProof (Brno, Czech Republic), with the GeneProof copy calculator (Brno, Czech Republic). One virus copy/mL was considered a positive result. Similarly, the tests mentioned above were used in the control group. No infection with the JC virus was detected in the group of patients infected with BKV.

### 4.3. Detection of Anti-EBV IgM and IgG Antibodies with the xMap Luminex Method

Sera of 27 patients were tested with the Epstein–Barr Virus Luminex xMAP test by Bio-Rad/Luminex (Toronto, Canada) in IgM and IgG classes. In the IgM class, antibodies against the VCA antigen were determined, and in the IgG class, the determined antibodies were against the VCA, EA-D and NA-1 antigens. The tests were carried out following the procedure provided by the manufacturer. The result is given in AU (arbitrary unit)/mL. The quantitative results were interpreted using the ATheNA computer programme from ZEUS Scientific (Branchburg, NJ, USA). The obtained results were interpreted according to the manufacturer’s protocol: <100 AU/mL—negative result, 100–120 AU/mL—borderline result, >120 AU/mL—positive result.

### 4.4. Detection of Anti-CMV IgM and IgG Antibodies with ELISA

Sera of 26 patients were tested with anti-CMV p52 ELISA (IgM) and anti-CMV gB ELISA (IgG) tests by EUROIMMUN (Lübeck, Germany). In the anti-CMV p52 ELISA (IgM) test, the CMV p52 antigen was used, which enabled a precise diagnosis of new infections, and in the anti-CMV gB ELISA (IgG) test, the CMV B glycoprotein (gB, UL55) was used to differentiate primary and past CMV infections. The obtained results were interpreted according to the manufacturer’s protocol: for IgM <0.8 RU (relative unit)/mL—negative result, 0.8–1.1 RU/mL—borderline result, ≥1.1 RU/mL—positive result; for IgG <16 RU/mL—negative result, 16–22 RU/mL—borderline result, ≥22 RU/mL—positive result. Results were read at 450 nm using a Biochrom Asys Expert 96 Microplate Reader (Biochrom Ltd., Cambridge, UK) and MicroWin 2000 S.C. Reader software (Biogenet, Jozefow, Poland).

### 4.5. Detection of Anti-Borrelia IgM and IgG Antibodies with Serological Methods

#### 4.5.1. IIFT

Sera from the experimental and control groups were tested with the anti-*Borrelia burgdorferi* sensu stricto IIFT test by EUROIMMUN (Lübeck, Germany) in the IgM and IgG classes. The test was performed and assessed according to the protocol prepared by the manufacturer, in which a positive reaction for the IgM class was detected at the initial dilution of 1:10 and in the IgG class at 1:100.

#### 4.5.2. ELISA

In the IgM and IgG classes, sera from the experimental and control groups were subjected to the anti-*Borrelia* ELISA screening test by EUROIMMUN (Lübeck, Germany). A complete panel of *B. burgdorferi* sensu stricto, *B. afzelii* and *B. garinii* antigens was used to evaluate the IgM class. In contrast, the full panel of *B. burgdorferi* sensu stricto, *B. afzelii*, *B. garinii* antigens and the recombinant protein VIsE of *B. burgdorferi* were used to assess the IgG class. According to the EUCALB requirements for interpreting results in LD diagnosis, as well as recommendations of the test manufacturer, sera with a value of ≥22 RU/mL were considered positive, those ranging between 16 and 22 RU/mL were considered borderline and those <16 RU/mL were regarded as negative [6,23]. Results were read at 450 nm using a Biochrom Asys Expert 96 Microplate Reader (Biochrom Ltd., Cambridge, UK) and MicroWin 2000 S.C. Reader software (Biogenet, Jozefow, Poland).

#### 4.5.3. IB

Regardless of the results obtained with the use of screening methods, the sera of the experimental and control groups were verified with the anti-*Borrelia* EUROLINE-RN-AT confirmation test by EUROIMMUN (Lübeck, Germany) in the IgM and IgG classes. The test used in the research is a third-generation serological test. In the IgM class, it contains the recombinant VIsE protein of *B. burgdorferi*, native antigens p41 (flagellin) and p39 (BmpA) belonging to *B. afzelii*, and OspC proteins belonging to four species: *B. afzelii*, *B. burgdorferi*, *B. garinii* and OspC *B. spielmanii*. In the IgG class, recombinant antigens were used: VIsE *B. burgdorferi*, *B. afzelii* and *B. garinii*; *B. burgdorferi* antigens: p18 (BB_P38), p19 (BB_N38), p20 (BB_Q03), p21 (BB_K53) and p58 (BB_A34); and native proteins: *B. burgdorferi* and *B. afzelii* lipid, *B. afzelii* p83, *B. garinii* p41 (flagellin), *B. garinii* p39 (BmpA) and *B. garinii* OspC. The tests were performed strictly according to the procedure specified by the test manufacturer. The results were interpreted with the application of a computer programme for analysis, EUROLineScan by EUROIMMUN (Lübeck, Germany).

### 4.6. Statistical Analysis

The statistical analysis of the obtained results was performed using Fisher’s exact test with R statistical package (STATISTICA version 3.1.3, Poland). Results *p* ≤ 0.05 were considered statistically significant.

## 5. Conclusions

The results of our research still show how important is the problem of false positive results in the serological diagnosis of LD. In addition, it confirms the correctness of using two-stage diagnostics with the WB or IB confirmatory test, which should become a mandatory test, regardless of the results obtained in the screening test. These studies also show the superiority of recombinant forms of antigens, which should encourage test manufacturers to look for more specific solutions such as characteristic fragments of protein sequences or their epitopes. Our results also indicate a significant potential for generating false positive results in serological tests used to diagnose LD in patients infected with EBV and CMV as well as with BKV.

## Figures and Tables

**Table 1:** Statistical parameters of Epstein–Barr virus (EBV), cytomegalovirus (CMV) and BK virus (BKV) copy numbers.

Statistical Parameters	EBV qPCR (Copies/mL) *n* = 27	CMV qPCR (Copies/mL) *n* = 26	BKV qPCR (Copies/mL) *n* = 16
Mean	1,190,912	3,608,075	2,569,704
SD	35,556,974	16,640,432	9,977,345
Min.	560	1078	920
Max.	17,800,568	85,093,861	39,976,524

**Legend:***n*, number of patients; qPCR, quantitative polymerase chain reaction; SD, standard deviation; Min., minimum value; Max., maximum value.

**Table 2:** The number and percentage of anti-*Borrelia* results obtained in the IgM and IgG class by enzyme-linked immunosorbent (ELISA), indirect immunofluorescence (IIFT) and immunoblot (IB) assays in patients infected with the Epstein–Barr virus (EBV), cytomegalovirus (CMV) and BK virus (BKV).

Virus	Type of Serological Test	Result	IgM *n* (%)	IgG *n* (%)
EBV *n* = 27	ELISA	POS/BOR	6 (22.2)	8 (29.6)
		NEG	21 (77.8)	19 (70.4)
	IIFT	POS/BOR	14 (51.9)	17 (63.0)
		NEG	13 (48.1)	10 (37.0)
	IB	POS/BOR	3 (11.1)	2 (7.4)
		NEG	24 (88.9)	25 (92.6)
CMV *n* = 26	ELISA	POS/BOR	2 (7.7)	1 (3.9)
		NEG	24 (92.3)	25 (96.1)
	IIFT	POS/BOR	4 (15.4)	4 (15.4)
		NEG	22 (84.6)	22 (84.6)
	IB	POS/BOR	6 (23.1)	3 (11.5)
		NEG	20 (76.9)	23 (88.5)
BKV *n* = 16	ELISA	POS/BOR	1 (6.2)	1 (6.2)
		NEG	15 (93.8)	15 (93.8)
	IIFT	POS/BOR	4 (25.0)	1 (6.2)
		NEG	12 (75.0)	15 (93.8)
	IB	POS/BOR	4 (25.0)	8 (50.0)
		NEG	12 (75.0)	8 (50.0)

NEG, negative result; BOR, borderline result; POS, positive result.

## Data Availability

Data are contained within the article.

## Supplementary Materials

The following are available online at https://www.mdpi.com/article/10.3390/pathogens11020203/s1, Table S1: detailed summary of results on determination of anti-*Borrelia* IgM and IgG antibodies obtained in serological tests in patients infected with the Epstein–Barr virus (EBV); Table S2: detailed summary of results on determination of anti-*Borrelia* IgM and IgG antibodies obtained in serological tests in patients infected with cytomegalovirus (CMV); Table S3: detailed summary of results on determination of anti-*Borrelia* IgM and IgG antibodies obtained in serological tests in patients infected with the BK virus (BKV); Table S4: detailed summary of results on determination of anti-*Borrelia* IgM and IgG antibodies obtained in serological tests in healthy individuals.
