# Supplementary material for: Cross-Reactive Results in Serological Tests for Borreliosis in Patients with Active Viral Infections
Source: Pathogens. 2022 Feb 3;11(2):203. doi: 10.3390/pathogens11020203 (PMC8879713; doi:10.3390/pathogens11020203)
Supplement: Supplementary file 1 [file pathogens-11-00203-s001.zip › pathogens-1553145-supplementary.pdf]

Table S1. Detailed summary of results on determination of anti-*Borrelia* IgM and IgG antibodies obtained in serological tests in patients infected with the Epstein-Barr virus (EBV).

No. of patient	EBV qPCR (copies/mL)	Anti-EBV xMap Luminex (AU/mL)				Anti-*Borrelia* IIFT (titre)		Anti-*Borrelia* ELISA (RU/ml)		Anti-*Borrelia* EUROLINE RN-AT IB - IgM								Anti-*Borrelia* EUROLINE RN-AT IB - IgG														
		IgM VCA	IgG EAD	IgG VCA	IgG NA1	IgM	IgG	IgM	IgG	VlsE B.b	p41 Flagellin B.a	p39 BmpA B.a	OspC B.a	OspC B.b	OspC B.g	OspC B.sp	Result	VlsE B.a	VlsE B.b	VlsE B.g	Lipid B.a	Lipid B.b	p83 B.a	p41 Flagellin B.g	p39 BmpA B.g	OspC B.g	p58 (BB_A 34)	p21 (BB_K 53)	P20 (BB_Q 03)	p19 (BB_N 38)	p18 (BB_P 38)	Result
1.	17,568	5	**10247**	**4006**	3	-	**1:100**	5.073	**18.248**	-	-	-	-	-	-	-	NEG	-	-	-	-	-	-	-	-	-	-	-	-	-	-	NEG
2.	2,367,893	**1098**	93	**1760**	43	**1:10**	-	5.097	8.810	-	**+**	-	**(+)**	**+**	**+**	-	**POS**	-	-	-	-	-	-	-	-	**(+)**	-	-	-	-	-	NEG
3.	27,690	33	**400**	**3371**	**514**	-	**1:100**	5.508	**68.595**	-	**(+)**	-	-	-	-	-	NEG	-	-	-	-	-	-	-	-	-	-	-	-	-	-	NEG
4.	17,800,568	**1075**	24	**1493**	15	**1:10**	-	**17.315**	5.982	-	-	-	-	-	-	-	NEG	-	-	-	-	-	-	-	-	-	-	-	-	-	-	NEG
5.	134,534	28	**235**	**560**	**222**	**1:10**	-	**24.115**	8.571	-	-	-	-	-	-	-	NEG	-	-	-	-	-	-	-	-	-	-	-	-	-	-	NEG
6.	4,235	51	**167**	**3880**	**325**	-	**1:100**	5.097	0.188	-	-	-	-	-	-	-	NEG	-	-	-	-	-	-	-	-	-	-	-	-	-	-	NEG
7.	1,003,456	18	**120**	**2333**	**498**	-	**1:100**	6.185	10.522	-	-	-	-	-	-	-	NEG	-	-	-	-	-	-	-	-	-	-	-	-	-	-	NEG
8.	5,467	40	**154**	**2669**	**487**	-	-	12.597	4.987	-	-	-	-	-	-	-	NEG	-	-	-	-	-	-	**+**	-	-	-	-	-	-	-	NEG
9.	75,430	52	**390**	**2853**	**446**	-	-	8.218	4.350	-	-	-	-	-	-	-	NEG	-	-	-	-	-	**+**	**(+)**	**+**	**+**	-	-	-	-	-	**POS**
10.	1,342	47	**385**	**3481**	**1205**	**1:10**	-	**186.688**	6.022	-	**+**	-	**(+)**	-	**+**	-	**POS**	-	-	-	-	-	-	-	-	-	-	-	-	-	-	NEG
11.	3,567,893	13	**530**	**1541**	**457**	-	**1:100**	3.016	**16.535**	-	-	-	-	-	-	-	NEG	-	-	-	-	-	-	-	-	-	-	-	-	-	-	NEG
12.	2,534	**190**	58	**3475**	33	**1:10**	**1:320**	4.976	9.168	-	-	-	-	-	-	-	NEG	-	-	-	-	-	-	-	-	-	-	-	-	-	-	NEG
13.	74,538	**188**	**176**	**4328**	**592**	**1:10**	**1:100**	1.636	4.549	-	-	-	-	-	-	-	NEG	-	-	-	-	-	-	-	-	-	-	-	-	-	-	NEG
14.	15,379	**460**	**(107)**	**3654**	42	**1:10**	-	1.927	4.190	-	**+**	-	-	-	-	-	NEG	-	-	-	-	-	**(+)**	**+**	-	-	-	-	-	-	-	NEG
15.	7,980	21	**284**	**3071**	**1399**	**1:10**	-	10.806	7.615	-	-	-	-	-	-	-	NEG	-	-	-	-	-	-	-	-	-	-	-	-	-	-	NEG
16.	1,238	40	**213**	**4378**	**1070**	**1:10**	**1:100**	**38.476**	5.106	-	-	-	-	-	-	-	NEG	-	-	-	-	-	-	-	-	-	-	-	-	-	-	NEG
17.	178,903	19	**354**	**4606**	**1930**	-	-	12.040	12.553	-	**+**	-	-	-	-	-	NEG	-	-	-	-	-	-	-	-	-	-	-	-	-	-	NEG
18.	1,212,346	**916**	29	**1234**	16	**1:10**	**1:100**	5.484	**45.297**	-	-	-	-	-	-	-	NEG	-	-	-	-	-	-	**+**	-	-	-	-	-	-	-	NEG
19.	5,430,987	**195**	36	**253**	37	**1:10**	-	**134.487**	**18.288**	-	-	-	-	-	-	-	NEG	-	-	-	-	-	**(+)**	**+**	**(+)**	-	-	-	-	-	-	NEG
20.	560	**919**	65	**2565**	40	**1:10**	**1:320**	7.298	8.173	-	**+**	-	-	-	-	-	NEG	-	-	-	-	-	-	**+**	-	-	-	-	-	-	-	NEG
21.	29,834	99	**323**	**3335**	**1133**	**1:10**	**1:100**	1.600	**17.093**	-	-	-	-	-	-	-	NEG	-	-	-	-	-	-	-	-	**(+)**	-	-	-	-	-	NEG
22.	3,854	**713**	**213**	**5108**	**209**	**1:10**	**1:100**	2.435	**17.929**	-	-	-	-	-	**(+)**	-	**BOR**	-	-	-	-	-	-	**(+)**	-	-	-	-	-	-	-	NEG
23.	17,345	**983**	46	**2339**	12	-	**1:100**	13.565	**18.009**	-	-	-	-	-	-	-	NEG	-	-	-	-	-	-	-	**+**	-	-	**+**	-	-	-	**POS**
24.	67,389	**680**	34	**2919**	62	-	**1:320**	**19.008**	9.088	-	-	-	-	-	-	-	NEG	-	-	-	-	-	-	**+**	**(+)**	**(+)**	-	-	-	-	-	NEG
25.	5,943	**514**	40	**3029**	64	-	**1:100**	4.976	5.027	-	-	-	-	-	-	-	NEG	-	-	-	-	-	-	**+**	-	-	-	-	-	-	-	NEG
26.	95,782	21	**(115)**	**1746**	**203**	-	**1:100**	4.250	9.168	-	**+**	-	-	-	-	-	NEG	-	-	-	-	-	-	-	-	-	-	-	-	-	-	NEG
27.	3,945	12	90	**2802**	**1188**	-	**1:100**	10.831	12.673	-	**+**	-	-	-	-	-	NEG	-	-	-	-	-	**(+)**	**+**	-	-	-	-	-	-	-	NEG

Legend:

qPCR - quantitative polymerase chain reaction

Anti-EBV xMap Luminex: <100 AU/mL - negative result, 100 - 120 AU/mL - borderline result, ≥120 AU/mL - positive result.

Anti-*Borrelia* indirect immunofluorescence test (IIFT): IgM ≥1:10 titer - positive result; IgG ≥1:100 titer - positive result.

Anti-*Borrelia* enzyme-linked immunosorbent assay (ELISA): Ratio <16 RU/mL - negative result, 16 - 22 RU/mL - borderline result, ≥22 RU/mL - positive result.

Anti-*Borrelia* EUROLINE RN-AT immunoblot (IB): „-" negative result, „(+)" – borderline result, „+" positive result.

Positive results are marked in bold and borderline ones are additionally marked in parentheses.

B.a — *Borrelia afzelii*, B.b — *Borrelia burgdorferi*, B.g — *Borrelia garinii*, B.sp — *Borrelia spielmanii*.

Table S2. Detailed summary of results on determination of anti-*Borrelia* IgM and IgG antibodies obtained in serological tests in patients infected with cytomegalovirus (CMV).

No. of patient	CMV qPCR (copies/mL)	Anti-CMV ELISA (RU/mL)		Anti-*Borrelia* IIFT (titre)		Anti-*Borrelia* ELISA (RU/ml)		Anti-*Borrelia* EUROLINE RN-AT IB - IgM								Anti-*Borrelia* EUROLINE RN-AT IB - IgG														
		IgM	IgG	IgM	IgG	IgM	IgG	VlsE B.b	p41 Flagellin B.a	p39 BmpA B.a	OspC B.a	OspC B.b	OspC B.g	OspC B.sp	Result	VlsE B.a	VlsE B.b	VlsE B.g	Lipid B.a	Lipid B.b	p83 B.a	p41 Flagellin B.g	p39 BmpA B.g	OspC B.g	p58 (BB_A 34)	p21 (BB_K 53)	P20 (BB_Q 03)	p19 (BB_N 38)	p18 (BB_P 38)	Result
1.	7,160	**1.3**	**34.45**	**1:10**	-	2.677	1.250	-	-	-	**+**	**+**	**+**	-	**POS**	-	-	-	-	-	-	-	-	-	-	-	-	-	-	NEG
2.	121,822	**6.6**	**>200**	-	-	0.545	3.115	-	-	-	-	-	**(+)**	-	**BOR**	-	-	-	-	-	-	**(+)**	-	**+**	-	-	-	-	-	**BOR**
3.	18,684	**2.1**	**85.34**	-	-	12.766	0.625	-	-	-	-	-	**(+)**	-	**BOR**	-	-	-	-	-	-	-	-	-	-	-	-	-	-	NEG
4.	3,160	**1.1**	**53.78**	-	-	3.210	3.075	-	**+**	-	-	-	-	-	NEG	-	-	-	-	-	-	-	**(+)**	-	-	-	-	-	-	NEG
5.	1,600	0.6	**48.01**	-	**1:100**	0.364	6.858	-	**+**	-	**(+)**	-	**(+)**	-	**BOR**	-	-	-	-	-	-	**+**	-	-	-	-	-	-	-	NEG
6.	3,040	**1.0**	**32.80**	-	-	1.636	3.314	-	-	-	-	-	-	-	NEG	-	-	-	-	-	-	-	-	-	-	-	-	-	-	NEG
7.	51,000	**4.1**	**93.87**	-	-	1.055	8.252	-	-	-	-	-	-	-	NEG	-	-	-	-	-	-	-	-	-	-	-	-	-	-	NEG
8.	1,937,095	**5.4**	**>200**	**1:10**	-	6.258	2.478	-	**+**	-	-	-	-	-	NEG	-	-	-	-	-	-	-	-	-	-	-	-	-	-	NEG
9.	1,078	0.2	**27.96**	-	-	**215.17**	0.969	-	-	-	**+**	**+**	**+**	**+**	**POS**	-	-	-	-	-	-	-	-	**+**	-	-	-	-	-	**BOR**
10.	51,842	**3.9**	**22.16**	-	-	1.200	1.219	-	-	-	-	-	-	-	NEG	-	-	-	-	-	-	-	-	-	-	-	-	-	-	NEG
11.	3,675,025	**3.3**	**>200**	-	-	2.605	4.628	-	-	-	-	-	-	-	NEG	-	-	-	-	-	-	**+**	-	-	-	-	-	-	-	NEG
12.	403,169	**5.7**	**21.91**	-	-	1.018	0.656	-	-	-	-	-	-	-	NEG	-	-	-	-	-	-	**(+)**	-	-	-	-	-	-	-	NEG
13.	2,900	0.3	**86.23**	-	-	0.836	4.628	-	-	-	-	-	-	-	NEG	-	-	-	-	-	-	**+**	-	-	-	-	-	-	-	NEG
14.	1,193	0.7	**34.56**	-	-	2.048	**20.154**	-	-	-	-	-	-	**(+)**	NEG	-	**+**	**+**	-	-	-	**+**	-	**+**	-	-	-	-	-	**POS**
15.	617,577	**8.1**	**132.23**	-	-	1.382	1.719	-	-	-	-	-	-	-	NEG	-	-	-	-	-	-	-	-	-	-	-	-	-	-	NEG
16.	5,126	**1.2**	**28.56**	-	-	1.018	2.040	-	-	-	-	-	-	-	NEG	-	-	-	-	-	-	-	-	-	-	-	-	-	-	NEG
17.	7,700	**1.3**	**27.54**	-	**1:100**	2.484	1.875	-	-	-	-	-	-	-	NEG	-	-	-	-	-	-	**(+)**	-	**(+)**	-	-	-	-	-	NEG
18.	97,687	**4.5**	**118.36**	-	**1:100**	0.618	7.257	-	-	-	-	-	-	-	NEG	-	-	-	-	-	-	**+**	-	-	-	-	-	-	-	NEG
19.	32,219	**1.6**	**95.05**	**1:10**	-	**111.41**	2.438	-	-	-	-	-	**(+)**	**+**	**POS**	-	-	-	-	-	-	**+**	-	-	-	-	-	-	-	NEG
20.	15,625	**0.8**	**74.03**	-	-	1.418	4.827	-	-	-	-	-	-	-	NEG	-	-	-	-	-	-	-	-	-	-	-	-	-	-	NEG
21.	69,770	**1.1**	**59.36**	-	-	0.655	1.500	-	-	-	-	-	-	-	NEG	-	-	-	-	-	-	-	-	-	-	-	-	-	-	NEG
22.	28,330	**1.4**	**35.62**	-	-	2.871	0.344	-	-	-	-	-	-	-	NEG	-	-	-	-	-	-	-	-	-	-	-	-	-	-	NEG
23.	1,505,279	**2.3**	**102.23**	-	**1:100**	2.411	4.708	-	-	-	-	-	-	-	NEG	-	-	-	-	-	-	**+**	-	-	-	-	-	-	-	NEG
24.	85,093,861	**4.3**	**184.34**	**1:10**	-	5.145	3.155	-	-	-	-	-	-	-	NEG	-	-	-	-	-	-	-	-	-	-	-	-	-	-	NEG
25.	13,114	**1.7**	**47.06**	-	-	1.600	5.425	-	-	-	-	-	-	-	NEG	-	-	-	-	-	-	**+**	-	-	-	-	-	-	-	NEG
26.	44,900	**3.3**	**25.21**	-	-	2.315	1.844	-	-	-	-	-	-	-	NEG	-	-	-	-	-	-	-	-	-	-	-	-	-	-	NEG

Legend:

qPCR - quantitative polymerase chain reaction

Anti-CMV IgM enzyme-linked immunosorbent assay (ELISA): Ratio <0.8 RU/mL - negative result, 0.8 - 1.1 RU/mL - borderline result, ≥1.1 RU/mL - positive result.

Anti-CMV IgG ELISA: Ratio <16 RU/mL - negative result, 16 - 22 RU/mL - borderline result, ≥22 RU/mL - positive result.

Anti-*Borrelia* indirect immunofluorescence test (IIFT): IgM ≥1:10 titer - positive result; IgG ≥1:100 titer - positive result.

Anti-*Borrelia* ELISA: Ratio <16 RU/mL - negative result, 16 - 22 RU/mL - borderline result, ≥22 RU/mL - positive result.

Anti-*Borrelia* EUROLINE RN-AT immunoblot (IB): „-" negative result, „(+)" – borderline result, „+" positive result.

Positive results are marked in bold and borderline ones are additionally marked in parentheses.

B.a — *Borrelia afzelii*, B.b — *Borrelia burgdorferi*, B.g — *Borrelia garinii*, B.sp — *Borrelia spielmanii*.

Table S3. Detailed summary of results on determination of anti-*Borrelia* IgM and IgG antibodies obtained in serological tests in patients infected with the BK virus (BKV).

No. of patient	BKV qPCR (copies/mL)	Anti-*Borrelia* IIFT (titre)		Anti-*Borrelia* ELISA (RU/ml)		Anti-*Borrelia* EUROLINE RN-AT IB - IgM								Anti-*Borrelia* EUROLINE RN-AT IB - IgG														
		IgM	IgG	IgM	IgG	VlsE B.b	p41 Flagellin B.a	p39 BmpA B.a	OspC B.a	OspC B.b	OspC B.g	OspC B.sp	Result	VlsE B.a	VlsE B.b	VlsE B.g	Lipid B.a	Lipid B.b	p83 B.a	p41 Flagellin B.g	p39 BmpA B.g	OspC B.g	p58 (BB_A 34)	p21 (BB_K 53)	P20 (BB_Q 03)	p19 (BB_N 38)	p18 (BB_P 38)	Result
1.	843,021	-	-	0.618	3.872	-	-	-	-	-	-	-	NEG	-	-	-	-	-	-	**+**	-	-	-	-	-	-	-	NEG
2.	12,170	-	-	0.836	2.080	-	-	-	-	-	-	-	NEG	-	-	-	-	-	-	**+**	-	-	-	-	-	-	-	NEG
3.	8,200	-	-	2.823	4.190	-	-	-	-	-	-	-	NEG	-	-	-	-	-	-	**+**	-	-	-	-	-	-	-	NEG
4.	15,802	**1:10**	-	**212.34**	**50.833**	-	**+**	-	**(+)**	-	**+**	**+**	**POS**	-	**(+)**	**(+)**	-	-	-	**+**	-	**+**	-	-	-	-	-	**POS**
5.	1,299	-	-	6.403	5.544	-	-	-	-	-	-	**(+)**	NEG	-	-	**+**	-	-	-	**+**	-	-	-	-	-	-	-	**BOR**
6.	8,802	-	**1:100**	2.750	6.022	-	-	-	**+**	**+**	-	-	**POS**	-	-	-	-	-	-	**+**	-	-	-	-	-	-	-	NEG
7.	920	-	-	0.655	0.625	-	-	-	-	-	-	-	NEG	-	-	-	-	-	-	**+**	-	-	-	-	-	-	-	NEG
8.	19,000	**1:10**	-	2.677	7.456	-	-	-	-	-	-	-	NEG	-	-	-	-	-	-	**+**	-	**+**	-	-	-	-	-	**BOR**
9.	8,930	-	-	0.327	2.836	-	-	-	-	-	-	-	NEG	-	-	-	-	-	-	**+**	-	-	-	-	-	-	-	NEG
10.	4,800	-	-	0.545	1.063	-	-	-	-	-	-	-	NEG	-	-	-	-	-	-	**+**	-	-	-	-	-	-	-	NEG
11.	16,718	**1:10**	-	2.169	2.717	-	-	-	-	-	-	-	NEG	-	-	-	-	-	-	**+**	-	**+**	-	-	-	-	-	**BOR**
12.	16,000	-	-	0.821	3.792	-	-	-	-	-	-	-	NEG	-	**(+)**	-	-	-	-	**(+)**	-	-	-	-	-	-	-	**BOR**
13.	3,100	**1:10**	-	2.266	1.906	-	-	-	-	-	-	-	NEG	-	-	-	-	-	-	-	**+**	-	-	**+**	-	-	-	**POS**
14.	39,976,524	-	-	7.105	8.929	-	-	-	**(+)**	**(+)**	**+**	-	**POS**	-	-	**(+)**	-	-	-	+	-	**+**	-	-	-	-	-	**BOR**
15.	174,384	-	-	1.782	4.469	-	-	-	-	-	-	-	NEG	-	-	-	-	-	-	+	-	-	-	-	-	-	-	NEG
16.	5,588	-	-	4.081	6.022	-	**(+)**	-	-	-	**+**	-	**POS**	-	-	-	-	-	-	+	+	**+**	-	-	-	-	-	**POS**

Legend:

qPCR - quantitative polymerase chain reaction

Anti-*Borrelia* indirect immunofluorescence test (IIFT): IgM ≥1:10 titer - positive result; IgG ≥1:100 titer - positive result.

Anti-*Borrelia* enzyme-linked immunosorbent assay (ELISA): Ratio <16 RU/mL - negative result, 16 - 22 RU/mL - borderline result, ≥22 RU/mL - positive result.

Anti-*Borrelia* EUROLINE RN-AT immunoblot (IB): „-" negative result, „(+)" – borderline result, „+" positive result.

Positive results are marked in bold and borderline ones are additionally marked in parentheses.

B.a — *Borrelia afzelii*, B.b — *Borrelia burgdorferi*, B.g — *Borrelia garinii*, B.sp — *Borrelia spielmanii*.

Table S4. Detailed summary of results on determination of anti-*Borrelia* IgM and IgG antibodies obtained in serological tests in healthy individuals.

No. of patient	EBV qPCR	CMV qPCR	BKV qPCR	Anti-EBV xMap Luminex (AU/ml)				Anti-CMV ELISA (RU/ml)		Anti-*Borrelia* IIFT (titre)		Anti-*Borrelia* ELISA (RU/mL)		Anti-*Borrelia* EUROLINE RN-AT IB - IgM								Anti-*Borrelia* EUROLINE RN-AT IB - IgG														
	(copies/mL)			IgM VCA	IgG EAD	IgG VCA	IgG NA1	IgM	IgG	IgM	IgG	IgM	IgG	VlsE B.b	p41 Flagellin B.a	p39 BmpA B.a	OspC B.a	OspC B.b	OspC B.g	OspC B.sp	Result	VlsE B.a	VlsE B.b	VlsE B.g	Lipid B.a	Lipid B.b	p83 B.a	p41 Flagellin B.g	p39 BmpA B.g	OspC B.g	p58 (BB_A 34)	p21 (BB_K 53)	P20 (BB_Q 03)	p19 (BB_N 38)	p18 (BB_P 38)	Result
1.	0	0	0	12	37	28	99	0.2	3	-	-	5.173	1.438	-	-	-	-	-	-	-	NEG	-	-	-	-	-	-	-	-	-	-	-	-	-	-	NEG
2.	0	0	0	45	5	46	**(105)**	0.6	**45**	**1:10**	-	4.087	1.610	-	-	-	-	-	-	-	NEG	-	-	-	-	-	-	-	-	-	-	-	-	-	-	NEG
3.	0	0	0	3	25	84	18	0,1	**68**	-	-	5.408	3.295	-	**(+)**	-	-	-	-	-	NEG	-	-	-	-	-	-	-	-	-	-	-	-	-	-	NEG
4.	0	0	0	27	35	28	**125**	0.02	2	-	-	7.315	5.982	-	-	-	-	-	-	-	NEG	-	-	-	-	-	-	-	-	-	-	-	-	-	-	NEG
5.	0	0	0	51	16	65	**159**	0.23	15	-	-	4.115	6.571	-	-	-	-	-	-	-	NEG	-	-	-	-	-	-	-	-	-	-	-	-	-	-	NEG
6.	0	0	0	17	9	38	23	0.21	7	-	**1:100**	5.097	0.188	-	-	-	-	-	-	-	NEG	-	-	-	-	-	-	-	-	-	-	-	-	-	-	NEG
7.	0	0	0	68	43	**(101)**	56	0.17	2	-	-	6.185	0.252	-	-	-	-	-	-	-	NEG	-	-	-	-	-	-	-	-	-	-	-	-	-	-	NEG
8.	0	0	0	75	36	74	**178**	0,59	8	-	-	2.897	4.627	-	-	-	-	-	-	-	NEG	-	-	-	-	-	-	-	-	-	-	-	-	-	-	NEG
9.	0	0	0	57	7	12	9	0.32	10	-	-	8.318	4.780	-	-	-	-	-	-	-	NEG	-	-	-	-	-	-	-	-	-	-	-	-	-	-	NEG
10.	0	0	0	8	38	65	**197**	0.43	**23**	**1:10**	-	6.158	1.022	-	**+**	-	-	-	-	-	NEG	-	-	-	-	-	-	-	-	-	-	-	-	-	-	NEG
11.	0	0	0	53	11	42	11	0.2	**(16)**	-	-	10.123	12.040	-	-	-	-	-	-	-	NEG	-	-	-	-	-	**(+)**	-	-	-	-	-	-	-	-	NEG
12.	0	0	0	42	19	59	65	0.32	**20**	-	-	4.378	1.956	-	-	-	-	-	-	-	NEG	-	-	-	-	-	-	-	-	-	-	-	-	-	-	NEG
13.	0	0	0	4	32	36	20	0.56	12	-	-	8.743	13.230	-	-	-	-	-	-	-	NEG	-	-	-	-	-	-	-	-	-	-	-	-	-	-	NEG
14.	0	0	0	27	45	74	47	0.11	**31**	-	-	1.005	3.567	-	-	-	-	-	-	-	NEG	-	-	-	-	-	-	-	-	-	-	-	-	-	-	NEG
15.	0	0	0	36	46	34	31	0.72	4	-	-	2.634	9.401	-	-	-	-	-	-	-	NEG	-	-	-	-	-	-	-	-	-	-	-	-	-	-	NEG
16.	0	0	0	12	63	56	42	0.17	15	-	**1:100**	7.835	**16.012**	-	**(+)**	-	-	-	-	-	NEG	-	-	-	-	-	-	-	-	-	-	-	-	-	-	NEG
17.	0	0	0	3	21	23	76	0.34	10	-	-	14.123	10.319	-	-	-	-	-	-	-	NEG	-	-	-	-	-	-	-	-	-	-	-	-	-	-	NEG
18.	0	0	0	56	27	2	35	0.55	14	-	-	12.105	7.324	-	**-**	**(+)**	-	-	**-**	-	NEG	-	-	-	-	-	-	-	-	-	-	-	-	-	-	NEG
19.	0	0	0	71	43	25	27	0.21	14	-	-	9.543	4.290	-	-	-	-	-	-	-	NEG	-	-	-	-	-	-	-	-	-	-	-	-	-	-	NEG
20.	0	0	0	23	29	39	43	0.34	**18**	-	-	8.675	2.402	-	-	-	-	-	-	-	NEG	-	-	-	-	-	-	-	-	-	-	-	-	-	-	NEG

Legend:

EBV - Epstein-Barr virus, CMV - cytomegalovirus, BKV - BK virus.

qPCR - quantitative polymerase chain reaction.

Anti-EBV xMap Luminex: <100 AU/mL - negative result, 100 - 120 AU/mL - borderline result, ≥120 AU/mL - positive result.

Anti-CMV IgM enzyme-linked immunosorbent assay (ELISA): Ratio <0.8 RU/mL - negative result, 0.8 - 1.1 RU/mL - borderline result, ≥1.1 RU/mL - positive result.

Anti-CMV IgG ELISA: Ratio <16 RU/ml - negative result, 16 - 22 RU/mL - borderline result, ≥22 RU/mL - positive result.

Anti-*Borrelia* indirect immunofluorescence test (IIFT): IgM ≥1:10 titer - positive result; IgG ≥1:100 titer - positive result.

Anti-*Borrelia* ELISA: Ratio <16 RU/mL - negative result, 16 - 22 RU/mL - borderline result, ≥22 RU/mL - positive result.

Anti-*Borrelia* EUROLINE RN-AT immunoblot (IB): „-" negative result, „(+)" – borderline result, „+" positive result.

Positive results are marked in bold and borderline ones are additionally marked in parentheses.

B.a — *Borrelia afzelii*, B.b — *Borrelia burgdorferi*, B.g — *Borrelia garinii*, B.sp — *Borrelia spielmanii*.
